# Functional Implications of MicroRNAs in Crohn’s Disease Revealed by Integrating MicroRNA and Messenger RNA Expression Profiling

**DOI:** 10.3390/ijms18071580

**Published:** 2017-07-20

**Authors:** Orazio Palmieri, Teresa Maria Creanza, Fabrizio Bossa, Tiziana Latiano, Giuseppe Corritore, Orazio Palumbo, Giuseppina Martino, Giuseppe Biscaglia, Daniela Scimeca, Massimo Carella, Nicola Ancona, Angelo Andriulli, Anna Latiano

**Affiliations:** 1 IRCCS ‘Casa Sollievo della Sofferenza’, Division of Gastroenterology, 71013 San Giovanni Rotondo, Italy; f.bossa@operapadrepio.it (F.B.); tiziana.latiano@gmail.com (T.L.); giuseppe.corritore@gmail.com (G.C.); giuseppina.martino@libero.it (G.M.); giuseppe.biscaglia@gmail.com (G.B.); danisci@hotmail.com (D.S.); a.andriulli@operapadrepio.it (A.A.); a.latiano@operapadrepio.it (A.L.); 2 Institute of Intelligent Systems for Automation, National Research Council, CNR-ISSIA, 70126 Bari, Italy; ancona@ba.issia.cnr.it; 3 Center for Complex Systems in Molecular Biology and Medicine, University of Turin, 10124 Turin, Italy; 4 IRCCS ‘Casa Sollievo della Sofferenza’, Division of Medical Genetics, 71013 San Giovanni Rotondo, Italy; o.palumbo@operapadrepio.it (O.P.); m.carella@operapadrepio.it (M.C.)

**Keywords:** Crohn’s disease, microRNA, mRNA, differential expression analysis, microRNA–mRNA co-expression

## Abstract

Crohn’s disease (CD) is a debilitating inflammatory bowel disease (IBD) that emerges due to the influence of genetic and environmental factors. microRNAs (miRNAs) have been identified in the tissue and sera of IBD patients and may play an important role in the induction of IBD. Our study aimed to identify differentially expressed miRNAs and miRNAs with the ability to alter transcriptome activity by comparing inflamed tissue samples with their non-inflamed counterparts. We studied changes in miRNA–mRNA interactions associated with CD by examining their differential co-expression relative to normal mucosa from the same patients. Correlation changes between the two conditions were incorporated into scores of predefined gene sets to identify biological processes with altered miRNA-mediated control. Our study identified 28 miRNAs differentially expressed (*p*-values < 0.01), of which 14 are up-regulated. Notably, our differential co-expression analysis highlights microRNAs (i.e., miR-4284, miR-3194 and miR-21) that have known functional interactions with key mechanisms implicated in IBD. Most of these miRNAs cannot be detected by differential expression analysis that do not take into account miRNA–mRNA interactions. The identification of differential miRNA–mRNA co-expression patterns will facilitate the investigation of the miRNA-mediated molecular mechanisms underlying CD pathogenesis and could suggest novel drug targets for validation.

## 1. Introduction

Crohn’s disease (CD) is a debilitating inflammatory bowel disease (IBD) characterized by a multifactorial aetiology, with complex interactions between a multitude of genes, gene products, epigenetic events, and environmental factors. Although over 200 genetic loci are associated with IBD [1], the genetic contribution of the majority of these loci toward the explained heritability of the disease is low [2]. Genetic analyses have successfully classified these genes as core components of several orderly biological mechanisms (e.g., autophagy, defective barrier function, IL-23 signalling, host-microbe interactions, and immune-mediated mechanisms). We corroborated and confirmed these data at the gene expression level, emphasizing the interaction between meta-omic and biological processes [3].

miRNAs may play a role in the induction of autoimmune diseases and IBD [4], and numerous studies have identified deregulated miRNAs in both tissue samples and sera collected from IBD patients [5,6,7,8,9,10,11,12,13,14,15]. However, the biological functions and functional targets of these miRNAs remain to be characterized. In IBD, single nucleotide polymorphisms (SNPs) located at miRNA binding sites can affect the expression of target messenger RNAs (mRNAs) involved in the pathogenesis of the disease. Brest et al. [16] have shown that a synonymous variant in the IRGM gene alters a binding site of miR-196, resulting in the deregulation of IRGM-dependent xenophagy in CD. In addition, miRNA-mediated dysregulation of IL-23R signaling is correlated with a single nucleotide polymorphism in the IL-23R gene and is strongly associated with IBD susceptibility [17]. For example, the interaction between miR-192 and the nucleotide-binding oligomerization domain-containing protein 2 (NOD2) gene may be relevant in the pathogenesis of IBD; SNP rs3135500 in the 3′-untranslated region (UTR) of NOD2 reduces the ability of miR-192 to inhibit NOD2 expression [18]. Fairfax et al. [19] have explored gene expression as a quantitative trait (eQTL mapping) for immunity-related genes and have suggested that specific risk alleles may alter gene expression profiles both locally (*cis*-acting) and at a distance (*trans*-acting).

Different genetic and epigenetic alterations may modify miRNA–mRNA interactions, thereby altering the co-expression profiles of miRNAs and mRNAs with or without changes in miRNA expression profiles [20].

Previous studies suggest that miRNA and mRNA expression data can be used to investigate differences in miRNA and mRNA expression as well as changes in miRNA–mRNA co-expression associated with CD pathogenesis. Within this framework, by using a new statistical data-driven approach, we aimed to predict the functional implications of miRNAs in CD by considering which pathways are altered by regulatory miRNA activity. We intended to capture changes in miRNA–mRNA co-expression resulting from genetic and epigenetic modifications that influence miRNA activity and, in turn, alter gene transcription.

The characterization of changes in global miRNA–mRNA networks will help to elucidate the miRNA-mediated molecular mechanisms underpinning CD physiopathology.

## 2. Results

Our study aims to predict the functional implications of microRNAs in CD by considering which pathways are affected by changes of regulatory miRNA activities. The idea is to capture alterations in miRNA-mRNA co-expressions resulting from genetic and epigenetic modifications that influence miRNA activity on gene transcription. 

To better understand the role of miRNAs in different biological processes, we designed a pathway enrichment analysis method based on the differential co-expression (DC) between miRNAs and mRNAs. The approach aims to highlight the pathways enriched for genes that change their functional interaction with a given miRNA or the entire miRNome in the two phenotypic conditions. The analysis is based on the comparison of miRNA-mRNA co-expression networks between inflamed CD tissues and matched non-inflamed mucosa. Correlation changes between the two conditions are incorporated into scores of predefined gene sets in order to identify signaling pathways and biological processes on which a miRNA exerts an altered regulatory activity. Specifically, the procedure searches for: (1) those miRNAs that are differentially co-expressed with the genes in a specific pathway, (2) pathways characterized by significant differences in co-expression with the entire assayed miRNome. We point out that this association with the phenotype is based on differences in the interaction between miRNAs and mRNAs including direct as well as mediated regulations.

### 2.1. Identification of Differentially Expressed Microrna

By comparing inflamed colonic mucosa from 15 CD patients with their non-inflamed controls, 103 of the 1105 unique miRNA probes were found to be differentially expressed (DE) with *p*-values < 0.05 (Table S1). Among these, 28 had a *p*-value < 0.01; in inflamed mucosa, 14 miRNAs (miR-193B, miR-19A, let-7I, miR-1273D, miR-886-5P, miR-668, miR-720, miR-455-3P, miR-3138, miR-612, miR-551B, miR-4264, let-7I-STAR, and miR-24) were upregulated, and the remaining 14 (miR-3194, miR-196A, miR-192, miR-200A, miR-192-STAR, miR-1913, miR-378B, miR-323B-3P, miR-3150, miR-422A, miR-611, miR-3184, miR-4284, and miR-129-STAR) were down-regulated (Table 1). Twelve of the 28 miRNAs (43%) were previously described as being associated with IBD: miR-196A [7], miR-192 [6,8,13], miR-193B [11], miR-19A [4], miR-200A [6], LET-7I [8], miR-455-3P, LET-7I-STAR, miR-422A [9], miR-24 [4,8,11], miR-1273D [13], and miR-4284 [13,14].

### 2.2. Identification of Molecular Pathways That Are Differentially Expressed and Differentially Co-Expressed with miRNAs

**Figure 1:**
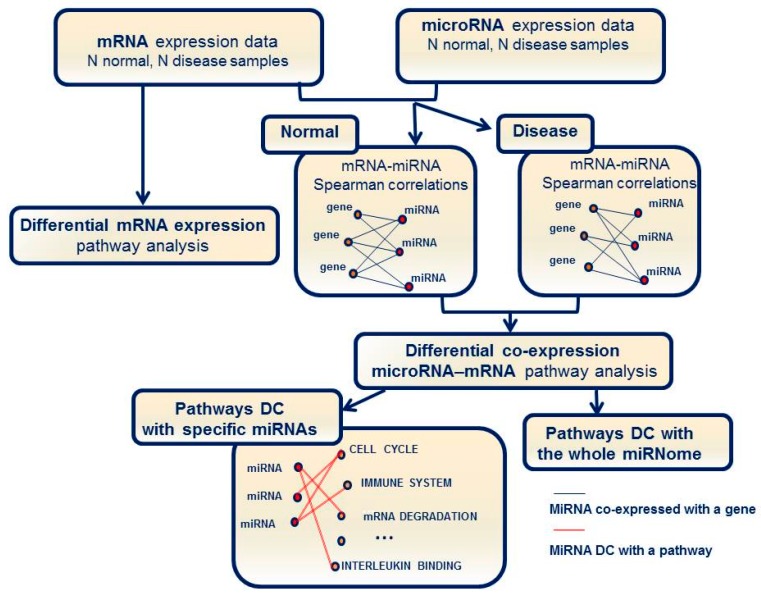
Schematic data and analysis workflow of differential expression (DE) in mRNA and miRNA as well as differential co-expression (DC) analyses.

Pathway analysis was performed as shown in Figure 1. We searched for differentially expressed mRNA pathways and for differentially co-expressed (DC) pathways (i.e., pathways characterized by significant changes in miRNA–mRNA interactions).

An analysis of the changes in miRNA–mRNA co-expression networks allowed us to isolate DC pathways within the miRNome (i.e., pathways enriched for genes that significantly change their co-expression with the entire miRNome). We foresee a relevant role for miRNAs in the deregulation of pathways identified as both DC and DE.

**Figure 2:**
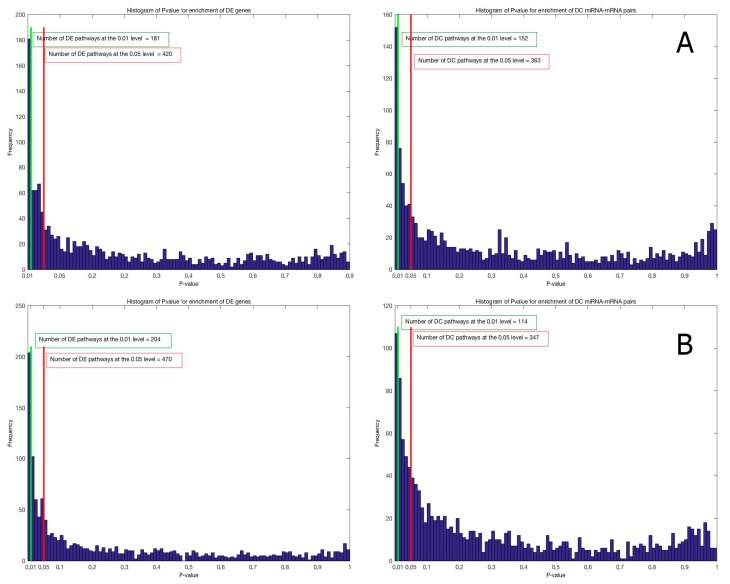
(**A**) Histogram of differentially expressed (DE) and differentially co-expressed (DC) enrichment *p*-values for the Gene Ontology term; (**B**) Histogram of differentially expressed (DE) and differentially co-expressed (DC) enrichment *p*-values for the canonical pathways.

DC and DE enrichment pathway analyses were performed on the C5 and C2Cp collections from the MSigDB database (http://software.broadinstitute.org/gsea/msigdb) version 5.0, and they include, respectively, 1454 lists of genes sharing a specific Gene Ontology (GO) term (Figure 2A) and 1330 gene sets belonging to canonical pathways from KEGG, BioCarta and Reactome (Figure 2B). The histograms in both Figure 2A,B show that there are significant differences in gene expression and miRNA–mRNA co-expression between IBD inflamed and not-inflamed tissues for the gene sets in the two analyzed collections. In each comparison, the number of significant gene sets at the 0.01 level is greater than the number expected by chance in the case of no significant change between the two conditions that is 15 and 13 for the GO terms and the canonical pathways, respectively.

**Figure 3:**
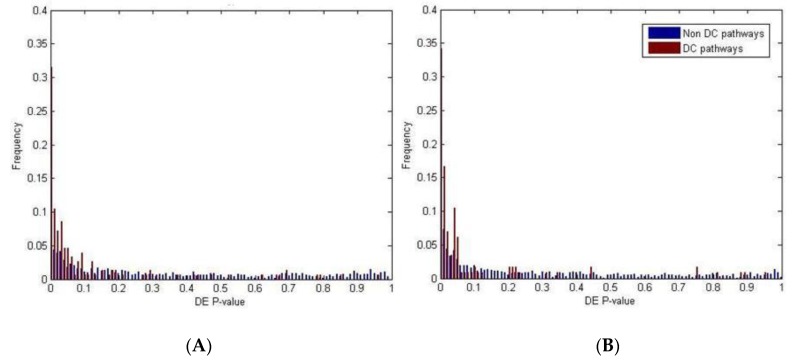
Frequencies of the differentially expressed (DE) *p*-values for the canonical pathways (**A**) and the Gene Ontology terms (**B**). The DE *p*-values for the differentially co-expressed (DC) pathways are plotted in red, while the DE *p*-values for the non-DC-pathways are plotted in blue. Most of the DC pathways have DE *p*-values < 0.05.

For canonical pathways, our analyses resulted in 204 DE and 114 DC pathways at the 0.01 level of significance; among them, 41 were both DE and DC (pathways indicated in bold in Table S2). For Gene Ontology terms, we identified 181 DE and 152 DC pathways at the 0.01 significance level: among them, 51 were both DE and DC (pathways indicated in bold in Table S3). In both collections, the overlap between the two lists was significant (Fisher’s exact test *p*-value = 10^−8^ for canonical pathways and *p*-value = 10^−13^ for Gene Ontology; Figure 3), suggesting that miRNAs have a significant biological impact on pathway deregulation.

**Figure 4:**
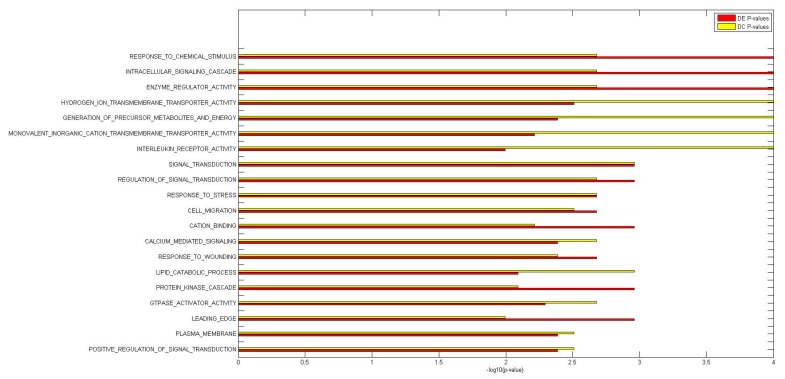
Histogram of the differentially expressed (DE) and differentially co-expressed (DC) −log10 *p*-value plots for the top 20 DC and DE GO terms.

**Figure 5:**
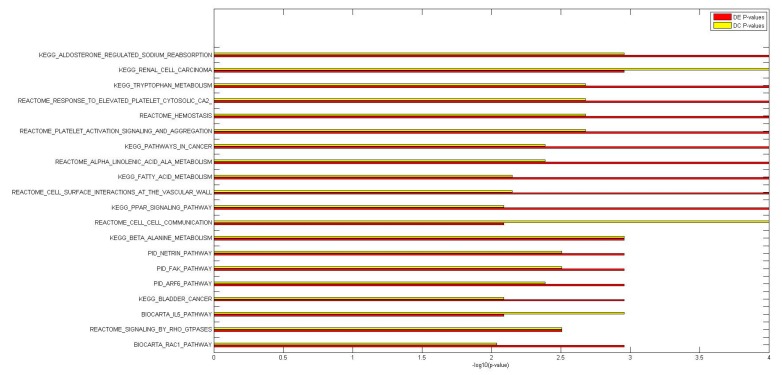
Histogram of differentially expressed (DE) and differentially co-expressed (DC) −log10 *p*-value plots for the top 20 DC and DE canonical pathways.

We concluded that miRNAs have a significant impact on the deregulation of biological processes and canonical pathways that have been implicated in IBD, a finding supported by genome-wide association studies (GWAS) of the response to a wide range of stressors (e.g., wounding and chemical or external stimuli), the nuclear factor-kappa B pathway, and GTPase and interleukin receptor activity (Tables S2 and S3 and Figure 4 and Figure 5). Specifically, among both DC and DE GO terms, we identified several biological mechanisms specifically involved in the response to a wide range of stressors: response to chemical stimulus (DE *p*-value = 10^−100^, DC *p*-value = 0.002), response to stress (DE *p*-value = 0.002, DC *p*-value = 0.002), response to wounding (DE *p*-value = 0.002, DC *p*-value = 0.004), and response to oxidative stress (DE *p*-value = 0.005, DC *p*-value = 0.005). DC and DE analyses of KEGG pathways suggested the involvement of these genes in leukocyte transendothelial migration (DE *p*-value = 0.004, DC *p*-value = 0.004).

### 2.3. Functional Implications of Specific miRNAs by Differential Co-Expression Pathway Analysis

Subsequently, we performed a more detailed differential co-expression pathway analysis: given a miRNA–mRNA pathway pair, we assessed the significance of pathway enrichment for the co-expression changes between genes in the pathway and a specific miRNA. We considered that a miRNA was DC with a pathway when the enrichment for this miRNA–mRNA pathway pair was associated with a *p*-value < 0.01.

Pathway analysis results are provided in the Tables S4 and S5, where all pathways are listed with the DE and DC enrichment *p*-values and FDR, together with the list of DC miRNAs.

**Figure 6:**
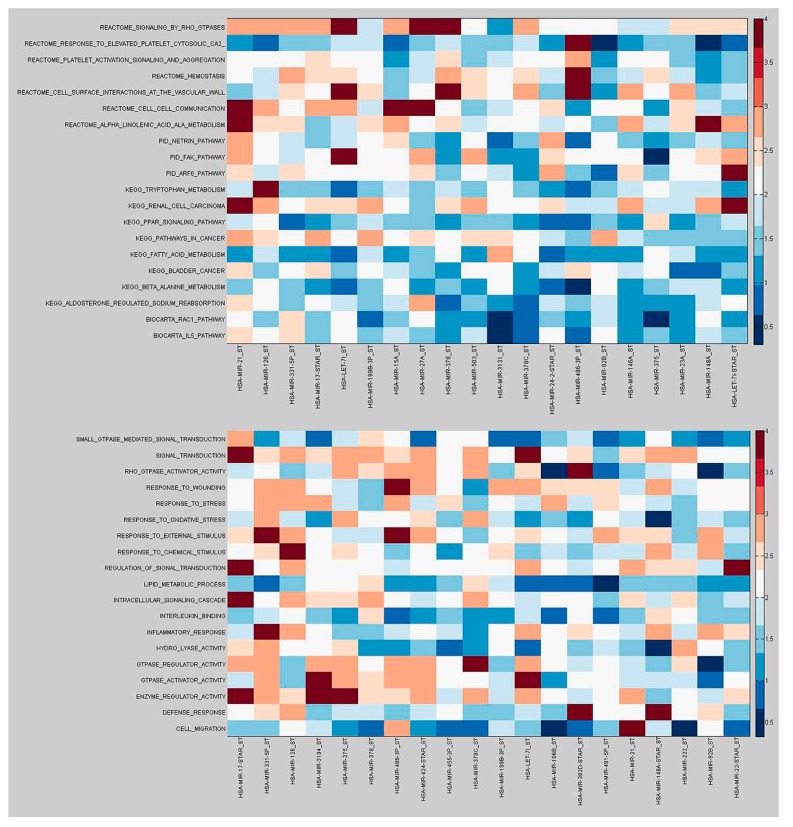
Heatmap of the –log^10^ (*p*-values) for differential co-expression between the top-ranked differentially co-expressed (DC) pathways, differentially expressed (DE) pathways, and specific miRNAs (top: Canonical pathways, bottom: GO terms).

Our analysis suggests the functional implications of a given miRNA by focusing on the interactions that are altered in the inflamed mucosa and, as a consequence, are important to understand the molecular mechanisms of the pathology in which the miRNA has a role (Figure 6).

For instance, our analysis indicates that miR-3194, the most down-regulated miRNA (*p* = 0.0005), was differentially co-expressed (*p* < 0.05) with all the GO term pathways shown in Figure 6, except “GTPase-mediated signal transduction” and “response to oxidative stress”. This perspective highlights a number of miRNAs that, although not DE, were DC with a great number of DE pathways. miR-21 was weakly deregulated in inflamed colonic mucosa and was co-expressed (*p* < 0.05) with all the C2Cp pathways shown in Figure 6 except “response to elevated platelet cytosolic CA2” and “fatty acid metabolism”.

In addition, by hypothesizing that miRNAs that were DC with differentially expressed pathways were miRNAs with regulatory activity on the whole pathway, we ordered the miRNAs for the number of DE pathways linked to them for differential co-expression. (Table 2 and Table S6). While, the list of the pathways differentially co-expressed with the top ranked miRNAs (*p* < 0.01) were shown in Table S7 (Canonical pathways) and in Table S8 (GO terms).

### 2.4. Differential miRNA–mRNA Co-Expression Highlights Known Inflammatory Bowel Disease miRNAs

Finally, we tested the global performance of our metric to reveal known IBD-associated miRNAs. We validated our results by comparing via statistical tests our findings with lists of miRNAs associated to the pathology by functional experiments that elaborate actions of individual miRNAs in known pathogenetic pathways in IBD as implicated by GWAS [21].

We found that the list of miRNAs with known functional interactions with key mechanisms implicated by GWAS in IBD is enriched of miRNAs differentially co-expressed with DE pathways. A Wilcoxon test shows that the recurrence frequencies in DE pathways of the IBD-associated miRNAs were significantly larger than those of random miRNA lists (C2Cp *p*-value = 0.005, GO terms *p*-value = 0.01). Notably, the list of these IBD miRNAs is not enriched by DE miRNAs (*p*-value = 0.23, Fisher’s exact test).

## 3. Discussion

Several studies have identified dysregulated miRNAs in patients with IBD, but little information is available on their functional roles in disease pathogenesis. In the present study, we analysed paired expression profiles of mRNAs and miRNAs in CD patients by comparing RNA from colonic mucosa with or without inflammation. 

Here, we report the possible role of miRNAs in CD using several methods. By differential expression analysis of miRNAs, and by examining gene co-expression networks. Moreover, to highlight miRNAs with the potential to act as causal regulators underlying changes in gene expression, we focused on the miRNAs that were differentially co-expressed with the most deregulated pathways. Our differential co-expression approach does not use any a priori information and thus presents several advantages compared with current miRNA–mRNA interaction scores. In particular, it allows for complete coverage of the assayed human genes, reduces bias due to incomplete knowledge obtained from the published literature, and it is able to infer condition-specific relationships [22]. This analysis complements our differential expression analysis to suggest a role in the disease onset for both differentially expressed miRNAs and miRNAs exhibiting significantly altered interactions with the most deregulated pathways in inflamed CD colonic mucosa.

Our analysis identified new dysregulated miRNAs (i.e., miR-866-5p and miR-668) that have been associated with bacterial infection. Specifically, miR-866-5p was increased in human monocyte-derived macrophages in response to several *Mycobacterium avium* subsp. *hominissuis* infection [23], while a significant increase in miR-668 was observed in circulation following lipoteichoic acid injection, a major component of the wall of gram-positive bacteria [24].

For pathways enriched for both differential mRNA expression and differential miRNA–mRNA co-expression, we can guess a relevant role for miRNAs in their deregulation. Among them, we found several biological mechanisms specifically involved in the response to a wide range of stress and to leukocyte transendothelial migration. These findings strengthen the importance of pathways involved in the response to “stress” or “wounding”, emphasizing the role of external triggers (environmental factors, including diet, luminal antigens or bacteria) in the induction of an exaggerated immune response.

A number of the canonical pathways that were both DE and DC were involved in the complex equilibrium of haemostasis. One, “platelet activation signaling and aggregation”, resulted the most differentially co-expressed pathway (DC *p*-value < 0.000001). The list of miRNAs that are involved in the deregulation of this pathway includes miR-21, miR-126, miR-146a, and miR-3194. There is evidence that the two main types of IBD, namely ulcerative colitis and CD, combine both inflammation and coagulation disorders in their pathogenesis and clinical manifestations. Accordingly, platelets emerge as key players in the inflammatory cascade [25]. Here, we underlined not only the roles of platelets in platelet-mediated inflammation in CD patients but also a critical role of miRNAs in the control of the mechanism of haemostasis.

A number of biological mechanisms, including “T and B cell proliferation”, “cytokine metabolic process”, “cytokine biosynthesis”, and “secretion” were found to be only differently co-expressed with miRNAs (*p* < 0.005). They have a significant role in immune system development; defects of primary epithelial aetiology lead to chronic, global gut inflammation, including dysfunction of innate immune responses and of epithelial barrier integrity [26,27,28]. Similarly, the “neurotransmitter release cycle” pathway and its sub-pathways (acetylcholine, serotonin, norepinephrine, glutamate and dopamine) were differently co-expressed with miRNAs (*p* < 0.002). It has been demonstrated that an intimate interaction between cells of the nervous and immune systems takes place in the gut and may have a role in diverse inflammatory disorders, such as CD [29].

Our analysis allowed us to identify DE miRNAs in the inflamed mucosa of patients with CD; these miRNAs target up hundreds of molecular pathways, resulting in the destabilization of biological processes. For instance, our analysis confirms the down-regulation of miR-4284 and suggests that it differentially regulates the genes responsible for the Janus kinase/signal transducers and activators of transcription (JAK-STAT) signaling. These results are consistent with earlier research that has shown the over-expression of this miRNA in cancer stem-like cells [30]. miR-21 is differentially co-expressed with the greatest number of differentially expressed canonical pathways (Figure 6). Among the top-ranked miRNAs in both the DE and DC analyses, miR-3194 is involved in targeting many dysregulated pathways, including those involved in the innate immune system. Specifically, miR-3194 mediates the regulation of signaling by interleukins (ILS), which are involved in cytokine signaling in the immune system. In addition, miR-3194 controls parts of two definite arms of the innate immune system: nucleotide binding domain leucine rich repeat containing receptor (NLR) signaling pathways, inflammasomes and the Nucleotide-Binding Oligomerization Domain, Leucine Rich Repeat and Pyrin Domain Containing 3 (NLRP3) inflammasome; and Toll receptor cascades and MYD88 signaling cascades initiated on the plasma membrane.

## 4. Materials and Methods

### 4.1. Patient Recruitment, Biopsy Collection and RNA Extraction

Fifteen CD patients diagnosed according to Lennard-Jones’ criteria [31] undergoing follow-up care at the IRCCS “Casa Sollievo della Sofferenza” Hospital (CSS), San Giovanni Rotondo, were considered eligible. The inclusion criteria, patient’s clinical characteristics and the procedures used for RNA extraction have been described previously [3]. In brief, the main inclusion criterion was an active flare-up of the disease, as defined by a Harvey–Bradshaw score >4. After written informed consent was obtained, specimens were collected from inflamed and adjacent (at least 30 cm away from the inflamed area) non-inflamed areas of the colon between 2011 and 2013. At the time of sample collection, 14 of the patients were not receiving medical treatment. Detailed baseline demographic characteristics and previous therapies have been described [3], and none of the patients received biological treatment.

The study and the experimental protocols were approved (N.12701/08; 10/10/2007) by the local Ethics committee of the IRCCS “Casa Sollievo della Sofferenza” Hospital, San Giovanni Rotondo, and were performed in accordance with the approved guidelines. A voluntary written informed consent was obtained from all participants, before study entry.

### 4.2. Microarray Analysis

The GeneChip Human Gene 1.0 ST Array System (www.affymetrix.com) interrogates 28,869 annotated genes with an average of 26 probes per gene. Each sample was processed as previously described by Palmieri et al. [3], The microarray data were deposited in ArrayExpress (www.ebi.ac.uk/arrayexpress), a public repository, under the accession number E-MTAB-2967.

The GeneChip miRNA 2.0 Array System (www.affymetrix.com) annotates 1105 mature miRNA probes from miRBase version 15 (http://microrna.sanger.ac.uk). A total of 500 ng of total RNA was labelled using the Genisphere HSR labelling kit (Affymetrix, Santa Clara, CA, USA) according to the manufacturer’s recommendations. First, poly(A) tailing was carried out at 37 °C for 15 min in a volume of 15 µL reaction mix containing 1× Reaction Buffer, 1.5 mL MgCl_2_ [25 mM], 1 mL ATP mix diluted 1:500 and 1 mL PAP enzyme. Second, FlashTag Ligation was performed at room temperature for 30 min by adding 4 mL of 5× FlashTag Ligation Mix Biotin and 2 mL T4 DNA Ligase into the 15 mL of reaction mix. To stop the reaction, 2.5 mL of Stop Solution was added. Samples were hybridized overnight to the Affymetrix Genechip miRNA 2.0 array and then washed and stained using standard Affymetrix protocols. The microarray data were deposited in ArrayExpress (www.ebi.ac.uk/arrayexpress) under the accession number E-MTAB-4371.

Both mRNA and miRNA expression profiles were evaluated by microarray at the CSS medical genetics facility.

### 4.3. Differential Expression Analysis of MicroRNAs and mRNAs

We obtained a total of 2N labelled specimens for the N subjects enrolled in the study, with N specimens belonging to the “normal/not inflamed” class and N to the “diseased” class. For each sample, the paired expression levels of G genes gi and M miRNAs mj were assayed for the two phenotypic conditions.

*p*-values for gene and miRNA differential expression were derived using two-tailed paired Student’s t-tests and were controlled for multiple testing by the Benjamini-Hochberg procedure [32].

### 4.4. Pathway Enrichment Analysis for Differential Expression

To assess evidence for the association of a given gene set with the phenotype, we computed gene set enrichment for differentially expressed genes and calculated a re-standardized *p*-value using a Random Set (RS) procedure [33,34]. Specifically, the statistical significance of the enrichment score was assessed with respect to two null hypotheses: the first concerns the lack of association between changes in gene expression and the phenotype; the second concerns the invariance of the enrichment score with respect to the identity of the genes in the gene set [32]. Here, we report a brief description of the Random Set procedure. More details can be found in Abatangelo et al. [34]. Let *s_i_*, with *i* = 1, 2, …, *G* be a score based on the two-sample *t*-statistic *t_i_* that measures the differential expression of the *i*-th gene between the two phenotypes or conditions. Specifically,

(1) $$s_{i}=\left|\Phi^{-1}\left(F\left(t_{i}\right)\right)\right|\;i=1,2,...,G,$$

where *F* is the cumulative distribution function for a *t* distribution having n-2 degrees of freedom, and Φ is the standard normal cumulative distribution function. Given a gene set composed of *K* genes, the re-standardized measure of its deregulation is,

(2) $$Z=\sqrt{\frac{K\left(G-1\right)}{G-K}}\frac{\bar{S}-\mu }{\sigma },$$

where $\bar{S}=\frac{1}{K}\sum_{i=1}^{K}s_{i}$, and *μ* and *σ* are the mean and the standard deviation, respectively, estimated based on the full set of gene scores. Significantly large values of *Z* are expected if the gene set is deregulated under the experimental conditions. The *p*-values were estimated using nonparametric permutation tests [35]. In particular, to test the first null hypothesis, the RS method performed 1000 random permutations of the phenotypic labels and recomputed the statistics for each shuffled data set. The re-standardization of the statistics was carried out to take the second test into account. The statistical significance of the Z score for each pathway analysed is controlled for multiple testing by the Benjamini-Hochberg false discovery rate (FDR) algorithm [32].

### 4.5. Pathway Enrichment Analysis for Differential Co-Expression between miRNAs and mRNAs

To unveil the role of miRNAs in different biological processes, we designed a pathway enrichment analysis method based on differential co-expression (DC) between miRNAs and mRNAs The method is based on a measure of correlation between the expression profiles of a gene *g_i_* and a miRNA *m_j_* assessed in the two phenotypic conditions. In particular, let $\rho_{i,j}$ and ${\tilde{\rho }}_{i,j}$ be the co-expressions of the gene *g_i_* and the miRNA *m_j_* in the normal and diseased samples, respectively. In our implementation, co-expressions were estimated using Spearman correlation coefficients. These estimates correspond to the linear Pearson correlations between ranks and consequently allow the detection of linear as well as non-linear interactions that are monotonic relationships between the variables. To quantify changes in co-expression between the gene *g_i_* and the miRNA *m_j_*, we introduced a DC score, which was defined as the absolute value of the difference between the co-expressions in the two analysed conditions. This is represented in the following formula:

(3) $$s_{i,j}=\left|T\left(\rho_{i,j}\right)-T\left(\tilde{\rho_{i,j}}\right)\right|,$$

where

(4) $$T\left(x\right)=\frac{1}{2}\sqrt{\frac{N-3}{1.06}}log\frac{1+x}{1-x}$$

is the Fisher transformation, which is adopted as an approximate variance-stabilizing transformation for the correlation values. Under the null hypothesis of absence of correlation, *T(x)* is distributed approximately as a standard normal. Given a pathway Γ composed of *K* genes, the change in the interaction of the miRNA *m_j_* with the entire set of genes is estimated by *Z_j_*(Γ), which is defined as,

(5) $$Z_{j}\left(\Gamma \right)=\sqrt{\frac{K\left(G-1\right)}{G-K}}\frac{{\bar{S}}_{j}-\mu }{\sigma }$$

where ${\bar{S}}_{j}=\frac{1}{K}\sum_{i=1}^{K}s_{i,j}$, and *μ* and *σ* are the mean and standard deviation, respectively, of $s_{i,j}$ estimated for the full set of genes. A random set procedure, as described in the previous section, is applied to assess the statistical significance of this score. The larger *Z_j_*(Γ), the more evidence for a role of the *j*-th miRNA in the deregulation of the pathway Γ: the pathway is associated with the disease when it is involved in significant changes in the interactions with miRNA. Note that since the $s_{i}$ score is normally distributed under the null hypothesis $\rho_{i,n}=0$, this method does not require transformation of the $s_{i}$ score as described in Equation (1) [32].

### 4.6. Pathway Enrichment Analysis for Differential Co-Expression Based on the Entire set of microRNAs 

A similar procedure was applied to infer deregulated pathways influenced by altered interactions with the entire set of miRNas (miRNome). In particular, enrichment pathway analysis was performed to assess evidence of the association of a pathway with the phenotype, as supported by changes in the interactions between the pathway and the entire miRNome. In this case, given a pathway Γ, the enrichment score is computed as follows:

(6) $$Z\left(\Gamma \right)=\sqrt{\frac{KM\left(GM-1\right)}{M(G-K)}}\frac{\bar{S}-\mu }{\sigma }$$

where $\bar{S}=\frac{1}{KM}\sum_{i=1}^{K}\sum_{j=1}^{M}s_{i,j}$, and *s_i,j_* is the score defined in Equation (3) related to the correlation between the *i-*th transcript and the *j-*th miRNA. Furthremore, *μ* and *σ* are the mean and the standard deviation, respectively, of $s_{i,j}$ estimated based on the complete sets of genes and miRNAs. The larger Z, the more evidence for an overall effect of miRNAs on the deregulation of the pathway. The random set procedure was carried out to assess the statistical significance of this enrichment score related to the entire miRNome.

Multiple hypothesis correction was addressed by controlling the false discovery rate associated with each *p*-value using the Benjamini-Hochberg correction [32]. The algorithms described herein were implemented in the Matlab programming language.

## 5. Conclusions

This analysis shows that our innovative procedure integrating miRNA and mRNA expression paired data, computing statistics at pathway level rather than at gene level, and exploiting the information of the biological pathways allows to better exploit expression data also in the case of small sample size. We concluded that our procedure highlights miRNAs that can suggest new biomarker candidates (some potentially druggable), providing novel hypotheses for specific functional experiments.

Our work suggests that analysing the expression of paired miRNA and mRNA data by investigating not only changes in miRNA and mRNA expression but also changes in miRNA–mRNA co-expression enhances the power of RNA analysis. Notably, the list of miRNAs linked to pathogenic pathways by GWAS is enriched for miRNAs that are differentially co-expressed with the most deregulated mRNA pathways but not for differentially expressed miRNAs. This demonstrates that considering information at the system level, such as differential co-expression measures between miRNAs and the deregulated pathways, allows increased focus on known causal miRNAs. Unveiling differential miRNA–mRNA co-expression properties gains insight into miRNA-mediated molecular mechanisms underlying the pathogenesis of disease and may suggest novel drug targets that can be validated.

## Figures and Tables

**Table 1:** Expression changes in the most deregulated microRNAs in Crohn’s disease. FC (fold change); STAR (antisense strand).

Gene Name	*p*-Value	FC
miR-3194	0.0005	−4.52
miR-196A	0.0005	−4.47
miR-192	0.001	−4.02
miR-193B	0.002	3.89
miR-19A	0.002	3.81
miR-200A	0.002	−3.75
miR-192-STAR	0.003	−3.65
LET-7I	0.003	3.64
miR-1273D	0.003	3.59
miR-886-5P	0.004	3.46
miR-668	0.004	3.45
miR-720	0.005	3.36
miR-455-3P	0.005	3.35
miR-1913	0.005	−3.32
miR-3138	0.005	3.29
miR-612	0.006	3.25
miR-378B	0.006	−3.22
miR-551B-STAR	0.006	3.22
miR-323B-3P	0.006	−3.22
miR-4264	0.006	3.22
LET-7I-STAR	0.007	3.16
miR-3150	0.007	−3.13
miR-422A	0.008	−3.12
miR-611	0.009	−3.04
miR-3184	0.009	−3.02
miR-4284	0.001	−3.00
miR-129-STAR	0.001	−3.00
miR-24	0.001	3.00

**Table 2:** Top-ranked miRNAs and the number of linked differential expression (DE) pathways for differential co-expression (DC).

miRNA	Number of DE Canonical Pathways	miRNA	Number of DE GO Terms
miR-21	45	miR-378	59
miR-27A	44	miR-187	58
miR-126	42	miR-3194	58
miR-3194	39	miR-126	55
miR-486-3P	39	miR-17-STAR	52
miR-148A-STAR	38	miR-378C	52
miR-378	38	miR-331-5P	50
miR-199B-3P	35	miR-623	49
miR-302D-STAR	35	miR-375	44
miR-3133	35	miR-3131	43
miR-378C	35	miR-378-STAR	43
miR-17-STAR	33	miR-486-3P	42
miR-187	33	LET-7I	41
miR-3131	33	miR-148A-STAR	41
miR-375	32	miR-422A	41
LET-7I	31	miR-1286	39
miR-623	29	miR-23A	39
miR-3120	28	miR-27A	39
miR-331-5P	26	miR-146A	38
miR-335-STAR	26	miR-222	37
miR-125B	24	miR-335-STAR	37
miR-199A-3P	24	miR-25	34
miR-223	24	miR-125B	32
miR-373-STAR	22	miR-200C	31
miR-22-STAR	21	miR-199B-3P	30
miR-323-5P	21	miR-363	30
miR-363	21	miR-21	29
miR-555	21	miR-4253	28

## Supplementary Materials

Supplementary materials can be found at www.mdpi.com/1422-0067/18/7/1580/s1.
