# Addendum: Palmieri, O. et al. Functional Implications of MicroRNAs in Crohn’s Disease Revealed by Integrating MicroRNA and Messenger RNA Expression Profiling. *Int. J. Mol. Sci.* 2017, *18*, 1580

**DOI:** 10.3390/ijms18102113

**Published:** 2017-10-09

**Authors:** Orazio Palmieri, Teresa Maria Creanza, Fabrizio Bossa, Tiziana Latiano, Giuseppe Corritore, Orazio Palumbo, Giuseppina Martino, Giuseppe Biscaglia, Daniela Scimeca, Massimo Carella, Nicola Ancona, Angelo Andriulli, Anna Latiano

**Affiliations:** 1 IRCCS ‘Casa Sollievo della Sofferenza’, Division of Gastroenterology, 71013 San Giovanni Rotondo, Italy; f.bossa@operapadrepio.it (F.B.); tiziana.latiano@gmail.com (T.L.); giuseppe.corritore@gmail.com (G.C.); giuseppina.martino@libero.it (G.M.); giuseppe.biscaglia@gmail.com (G.B.); danisci@hotmail.com (D.S.); a.andriulli@operapadrepio.it (A.A.); a.latiano@operapadrepio.it (A.L.); 2 Institute of Intelligent Systems for Automation, National Research Council, CNR-ISSIA, 70126 Bari, Italy; creanza@ba.issia.cnr.it (T.M.C.); ancona@ba.issia.cnr.it (N.A.); 3 Center for Complex Systems in Molecular Biology and Medicine, University of Turin, 10124 Turin, Italy; 4 IRCCS ‘Casa Sollievo della Sofferenza’, Division of Medical Genetics, 71013 San Giovanni Rotondo, Italy; o.palumbo@operapadrepio.it (O.P.); m.carella@operapadrepio.it (M.C.)

The authors would like to indicate that Dr. Orazio Palmieri and Dr. Teresa Maria Creanza contributed equally to the work published in the International Journal of Molecular Sciences [1]. For this reason both should be considered as co-first authors. The authors apologize for any inconvenience this change may cause.

The changes do not affect the scientific results. The manuscript will be updated and the original will remain online on the article webpage.
