# Supplementary material for: Functional Implications of MicroRNAs in Crohn’s Disease Revealed by Integrating MicroRNA and Messenger RNA Expression Profiling
Source: Int J Mol Sci. 2017 Jul 20;18(7):1580. doi: 10.3390/ijms18071580 (PMC5536068; doi:10.3390/ijms18071580)
Supplement: Supplementary file 1 [file ijms-18-01580-s001.zip › ijms-207430-supplementary/Supplementary Table legends_palmieri.pdf]

Supplementary Table legends:

Supplementary table 1. Expression changes in deregulated miRNAs in Crohn's disease, p-value <0.05. FC, fold change.

Supplementary table 2: Canonical pathways differently co-expressed with miRNAs. Pathways in bold were both differently expressed (DE) and differently co-expressed (DC).

Supplementary table 3: Gene Ontology pathways differently co-expressed with miRNAs. Pathways in bold were both differently expressed (DE) and differently co-expressed (DC).

Supplementary table 4: Differential co-expression pathway analysis in the canonical pathways. miRNAs that were DC with a pathway enriched for this miRNA-mRNA pathway pair were associated with a P-value < 0.01.

Supplementary table 5: Differential co-expression pathway analysis in Gene Ontology pathways. miRNAs that were DC with a pathway enriched for this miRNA-mRNA pathway pair were associated with a P-value < 0.01.

Supplementary table 6: miRNAs ordered for the number of linked DE pathways for differential co-expression (DC).

Supplementary table 7: Differential co-expression (DC) pathway analysis for the canonical pathways. Lists of pathways that were DC with each miRNA with a P-value < 0.01.

Supplementary table 8: Differential co-expression (DC) pathway analysis for the GO terms. Lists of terms that were DC with each miRNA with a P-value < 0.01.
